# Childhood family environment and μ-opioid receptor availability in vivo in adulthood

**DOI:** 10.1038/s41386-025-02059-6

**Published:** 2025-01-31

**Authors:** Aino Saarinen, Lauri Tuominen, Sampsa Puttonen, Olli Raitakari, Liisa Keltikangas-Järvinen, Jarmo Hietala

**Affiliations:** 1 Department of Psychology, Faculty of Medicine, University of Helsinki, Helsinki, Finland; 2 Turku PET Centre and Turku University Hospital, University of Turku, Turku, Finland; 3 Department of Psychiatry, University of Turku and Turku University Hospital, Turku, Finland; 4 Institute of Mental Health Research, Royal Ottawa Mental Health Centre, Ottawa, ON Canada; 5 Faculty of Social Sciences, Tampere University, Tampere, Finland; 6 Research Centre of Applied and Preventive Cardiovascular Medicine, Faculty of Medicine, University of Turku, Turku, Finland; 7 Centre for Population Health Research, University of Turku and Turku University Hospital, Turku, Finland; 8 Department of Clinical Physiology and Nuclear Medicine, Turku University Hospital, Turku, Finland

**Keywords:** Social neuroscience, Developmental biology, Human behaviour

## Abstract

Animal studies have reported associations of early maternal separation with altered μ-opioid receptor function but data on humans are scarce. We now investigated whether childhood family environment is related to μ-opioid receptor availability in the human brain in adulthood. Healthy participants (*n* = 37–39 in the analyses) were recruited from the prospective population-based Young Finns Study (YFS) that started in 1980. Childhood family environment was evaluated in 1980, including scores for stress-prone life events, disadvantageous emotional family atmosphere, and adverse socioeconomic environment. We used positron emission tomography (PET) with radioligand [^11^C]carfentanil to measure μ–opioid receptor availability in adulthood. Age- and sex-adjusted analyses showed that exposure to stress-prone life events in childhood was related to lower μ-opioid receptor binding in the orbitofrontal cortex, hippocampus, putamen, amygdala, insula, thalamus, anterior cingulate cortex, and dorsal caudate in adulthood (when compared to participants not exposed to stress-prone life events). Unfavorable socioeconomic family environment or disadvantageous emotional family atmosphere was not associated with μ-opioid receptor availability in adulthood. In conclusion, exposure to environmental instability (i.e., to stress-prone life events below traumatic threshold) during early development is associated with dysregulation of the u-opioid receptor transmission in adulthood. The findings increase understanding of the neurobiological mechanisms involved in the associations between childhood adversities and adulthood mental disorders.

## Introduction

Early life adversities, such as parental neglect, abuse, violence, or poverty, are present in a third of individuals with psychiatric disorders in adulthood [1, 2]. Exposure to adverse childhood experiences is shown to elevate risk of, for example, major depressive disorder, generalized anxiety disorder, substance abuse, and schizophrenia [1, 3–7]. While a correlation between adverse childhood circumstances and adulthood mental disorders is well-documented, the mechanisms are not yet fully established.

One mechanism between childhood adversities and mental disorders could be alterations in the endogenous opioid system in the brain. The opioid system is involved in reward processing [8], pleasure in social interaction [9, 10], experiences of affective pain [11]. Animal studies have reported that early maternal separation is related to minor changes in κ- or δ-opioid receptor density in rats [12, 13]. Additionally, offspring rats exposed to maternal separation are reported to have a stronger preference for morphine [14] and more evident reductions in negative emotions after buprenorphine administration [15] when compared to offspring without early social trauma. Moreover, it has been suggested that mu opioid receptors play a role in separation-induced distress [16]: for example, morphine reduces but naloxone increases separation distress in guinea pigs [17]. Taken together, evidence from experimental studies provides support for a hypothesis that childhood separations may predict life-long alterations in the endogenous opioid system (Preter & Klein, 2014).

Also, candidate-gene studies have implied a role for the opioid system in the mechanisms between early adversities and later psychosocial adjustment. That is, a μ-opioid receptor gene (OPRM1) is found to interact with parenting practices (in terms of parental monitoring, early maternal care, or parental attention) when predicting the likelihood of alcohol use disorders [18], fearful attachment [19], or seeking for social support during stress [20].

As far as we know, however, only one previous study has investigated the association of childhood circumstances with functioning of the opioid system system in vivo. The study did not find any association between retrospectively evaluated childhood adversities and mu opioid receptor availability in healthy volunteers [21].

In the current study, we prospectively investigated whether qualities of childhood family environment are related to functioning of the opioid system in adulthood in the human brain. This study used a dataset that was originally collected to examine the role of brain opioid system in the temperament trait Harm Avoidance [22]. We used positron emission tomography (PET) with radioligand [^11^C]carfentanil to measure μ–opioid receptor availability (*n* = 37–39). Family environment was referred in terms of (1) adverse socioeconomic environment (i.e., parents’ low occupational status or low educational level, low family income in relation to family size, unstable employment situation, over-crowded apartment), (2) disadvantageous emotional family atmosphere (i.e., parent’s mental disorder, parent’s frequent alcohol intoxication, emotional distance between the child and parent, parental intolerance toward the child, parental life dissatisfaction), and (3) stress-prone life events (i.e., change of residence or school, parental divorce, parent’s death, parent’s hospitalization, child’s long-term absence from school due to sickness).

## Materials and methods

### Participants

This study was a part of the ‘Neurobiology of Personality’ project at the University of Turku and University of Helsinki (Finland). The same dataset has been used also previously in Tuominen’s et al. studies [22–24]. The participants for this study (n = 39) were selected from the prospective population-based Young Finns Study (YFS) that started in 1980 (n = 3596 in the baseline study, born in 1962, 1965, 1968, 1971, 1974, 1977). The original sampling of YFS was designed to include a population-based sample of non-institutionalized Finnish children, representative with regard to Eastern vs. Western regions in Finland, sex (female vs. male), and rural vs. urban environment.

For the present sub-study, we utilized data that were originally collected to examine the role of brain opioid system in the temperament trait Harm Avoidance [22]. The participants were selected from the YFS sample on the basis of their Harm Avoidance scores (HA, a scale of the Temperament and Character Inventory). In the current study, we invited all the participants with low/high HA who could be matched with each other with regard to age, sex, and educational level. Further details about the sampling can be found elsewhere [22, 23, 25].

All participants were screened to be healthy on the basis of blood and urine screening, medical examinations and interviews, MRI, and ECG examination. An extensive urine drug screen was conducted, and alcohol use was assessed with the AUDIT questionnaire and blood laboratory tests. Participants were allowed to have past affective disorders, but none of them fulfilled diagnostic criteria at the time of PET imaging (six participants had mild/moderate depressive or anxiety symptoms). According to the Hamilton Depression Rating Scale (HAM-D-17), all participants had scores <10. Participants with regular smoking were excluded because smoking is known to affect [^11^C]carfentanil binding potential [26].

The study was conducted in accordance with the Declaration of Helsinki. The original design of the YFS was approved by the ethical committees of all the Finnish universities with medical schools. Further, the current study protocol was approved by the Joint Ethical Committee of the University of Turku and the Turku University Central Hospital. All the participants gave a written informed consent before participation.

### Childhood environmental characteristics

Childhood family environment was assessed with three scores: (1) stress-prone childhood events, (2) disadvantageous emotional family atmosphere, and (3) adverse socioeconomic environment. The risk scores have been used and validated previously [27, 28]. All the childhood environmental characteristics were assessed with questionnaires presented for the parents in 1980.

*The score of adverse socioeconomic environment* included parents’ low occupational status (1 = highest parental occupational status was manual worker, 0 = at least one parent had lower- or higher-level non-manual occupation), parents’ low educational level (1 = parents’ highest completed education was comprehensive school, 0 = at least one parent had completed high school, occupational school, or academic level), low family income in relation to family size (1 = at least 1 SD below the sample average, 0 = other values), unstable employment situation (1 = at least one parent was unemployed or in a long-term sick leave, 0 = other employment situations), and over-crowded apartment (1 = number of rooms at home in relation to family size was at least 1 SD below the sample average, 0 = other values). We calculated a total score of adverse socioeconomic environment and dichotomized it (1 = at least one socioeconomic risk factor, 0 = no socioeconomic risk factors).

*The score of stress-prone childhood events* included the following factors: change of residence (at least once), change of school (at least once), parental divorce, mother’s or father’s death, mother’s or father’s hospitalization (for at least one day), and child’s absence from school due to sickness (at least 11 days during past 12 months). Each event was dichotomously encoded (0 = not occurred, 1 = occurred). We calculated a sum score of the stressful life events and classified it as 0 (no stress-prone life events) or 1 (at least one stress-prone life event).

*The score of disadvantageous emotional family atmosphere* included mother’s or father’s mental disorder (0 = none of the parents had mental disorder, 1 = at least one parent had mental disorder), mother’s or father’s frequent alcohol intoxication (0 = parents reported alcohol intoxication at most once a week, 1 = at least one parent reported alcohol intoxication at least two times a week), emotional distance between the child and parent (0 = emotional closeness between the parent and child, 1 = emotional detachment between the parent and child), parental intolerance toward the child (0 = no parental intolerance toward the child, 1 = parental intolerance toward the child), and parental life dissatisfaction (0 = no parental dissatisfaction, 1 = parental dissatisfaction at least one life sector). We calculated a total score of disadvantageous emotional family atmosphere in childhood and dichotomized it (0 = no emotional risk factors, 1 = at least one emotional risk factor).

More specifically, emotional distance between the parent and child was evaluated with a four-item questionnaire (e.g., “The child is emotionally important for me”, ”I can fulfill myself with the child”). The items were responded with a 5-point scale (e.g., 1 = little, 5 = much). Emotional detachment between the parent and child was defined to be present if the parent responded to at least one item with either of the two most unfavorable response alternatives. Parental intolerance toward the child was evaluated with a three-item scale (“I get nervous when spending time with the child”, “The child is a burden in challenging situations”, “The child consumes my time too much”). The items were responded with a 5-point scale (1 = frequently, 5 = never). Parental intolerance toward the child was defined to be present if the parent responded to at least one item with “frequently” or “quite frequently”. The items measuring parenting attitudes have been used also previously [29, 30].

Parental life satisfaction was assessed with a three-item questionnaire measuring parent’s satisfaction in three life sectors: as a parent, spouse, and employee. The items were responded with a 5-point scale (1 = satisfied, 5 = dissatisfied). This questionnaire has been adapted from the Operation Family Study questionnaire [31] and has been used also previously [32, 33]. Parental dissatisfaction was defined to be present if parent reported being “dissatisfied” or “quite dissatisfied” in at least one of the life sectors.

### Sensitivity analyses

We conducted additional sensitivity analyses using Harm Avoidance and adult attachment style as covariates since they are previously found to associate with μ-opioid receptor availability [22, 24].

Harm Avoidance was measured in 2012 with the Harm Avoidance scale of the Temperament and Character Inventory (TCI) [34]. The scale includes 35 items that are responded with a 5-point scale (1 = totally disagree; 5 = totally agree). The internal consistency of the scale was good (Cronbach’s alpha = 0.96). We calculated a sum score of the items for all the participants who had responded to at least 50% of the items.

Adult attachment style was assessed using the Adult Attachment Interview (AAI) [35] that was further encoded using the Dynamic Maturational Model (DMM) [36]. The DMM has been widely used also previously [24, 37, 38]. The assessment of adult attachment style was conducted by an experienced AAI interpreter (A. H.) with qualified training on both AAI and DMM. Adult attachment style was categorized into three categories: avoidant (*n* = 15), ambivalent (*n* = 19), secure attachment (*n* = 15). More details about the assessment of adult attachment can be found elsewhere [24].

### PET imaging and processing

All participants underwent a PET scan with μ–opioid receptor tracer [^11^C]carfentanil as described in detail in [22]. [^11^C]carfentanil (dose 423.6 ± 73.9 MBq; mass 1.08 ± 0.84 μg) was injected as an intravenous bolus. A brain‐dedicated high‐resolution PET scanner (ECAT HRRT, Siemens Medical Solutions) was used for PET imaging collect emission data for 69 min using 16 frames (3 × 1 min, 4 × 3 min, and 9 × 6 min). During the PET scans, head of the subject was fixed using an individually molded thermoplastic mask. A T1‐weighted MRI scan with 1 × 1 × 1 mm^3^ resolution voxel size was obtained from each subject using Philips Gyroscan Intera 1.5 T CV Nova Dual MRI scanner to exclude structural abnormalities and for anatomical reference.

PET images were preprocessed using the automated PET data processing pipeline Magia [39] (https://github.com/tkkarjal/magia) running on MATLAB (The MathWorks, Inc., Natick, Massachusetts, United States). PET data was first corrected for motion by realigning the frames of each scan. Radiotracer binding was quantified using non-displaceable binding potential (*BP*_ND_), which is the ratio of specific binding to non-displaceable binding in the tissue [40]. The *BP*_*ND*_ is taken here as an estimate for number of target receptor/transporter available for tracer binding (receptor availability). Binding potential was calculated applying basis function method for each voxel using the simplified reference tissue model [41], with occipital cortex serving as the reference region. The parametric images were spatially normalized to MNI-space via segmentation and normalization of T1-weighted anatomical images, and finally smoothed with an 8-mm full-width half maximum Gaussian kernel.

The data were analyzed by averaging *BP*_ND_’s within regions of interest (ROIs). Atlas-based ROIs were generated in the brain regions rich with μ–opioid receptors (amygdala, hippocampus, ventral striatum, dorsal caudate, thalamus, insula, prefrontal cortex, orbitofrontal cortex, and anterior cingulate cortex using AAL [42] and Anatomy [43] toolboxes.

### Statistical analysis

Data were analyzed with STATA MP 16.0 statistical software with general linear models. Mean regional [^11^C]carfentanil was extracted for each region and was predicted separately by three dichotomous variables: 1) stress-prone life events (0 = no stressful life events, 1 = at least one stressful life event), 2) disadvantageous emotional family atmosphere (0 = no disadvantageous emotional factors, 1 = at least one disadvantageous emotional factor in childhood environment), and 3) adverse socioeconomic environment (0 = no unfavorable socioeconomic factors, 1 = at least one unfavorable socioeconomic factor). Since age and sex affect [^11^C]carfentanil *BP*_ND_ [44], they were used as covariates. In further sensitivity analyses, we used Harm Avoidance and adult attachment style as covariates because Harm Avoidance and adult attachment style are found to correlate with μ-opioid receptor availability in the same dataset [22, 24]. We reported both uncorrected p-values and false discovery (FDR) corrected p-values (Benjamini-Hochberg procedure) [45].

## Results

Participants were on average 37.4 years old (*SD* = 4.81) and 19 (48.7%) of them were female. The frequencies of different childhood adversities are shown in Table 1. Altogether 15 (40.5%) participants had been exposed to at least one stress-prone life event, 12 (31.6%) participants to at least one disadvantageous emotional factor, and 19 (48.7) participants to at least one adverse socioeconomic factor in childhood. The tetrachoric correlations between the three composite scores were weak [*r*(emotional score and socioeconomic score) = −0.13, *r*(emotional score and life event score = −0.01), *r*(socioeconomic score and life event score) = 0.02].

**Table 1:** Descriptive statistics of the childhood adversities.

	Frequency (%)
At least one stress-prone life event	15 (40.5)
Change of residence	11 (28.2)
Change of school	2 (5.3)
Parental divorce	3 (7.7)
Parental death	0 (0.0)
Mother’s hospitalization	2 (5.1)
Father’s hospitalization	1 (2.6)
Long-term absence from school	6 (15.8)
At least one disadvantageous emotional characteristic	12 (31.6)
Parental mental disorder	1 (2.6)
Frequent parental alcohol intoxication	2 (5.1)
Emotional detachment with parent	5 (12.8)
Parental intolerance	4 (10.3)
Parental life dissatisfaction	4 (10.5)
At least one unfavorable socioeconomic factor	19 (48.7)
Low parental occupational level	14 (35.9)
Low parental educational level	10 (25.6)
Low family income in relation to family size	7 (18.0)
Unstable parental employment situation	1 (2.6)
Over-crowded apartment	5 (12.8)

**Fig. 1:**
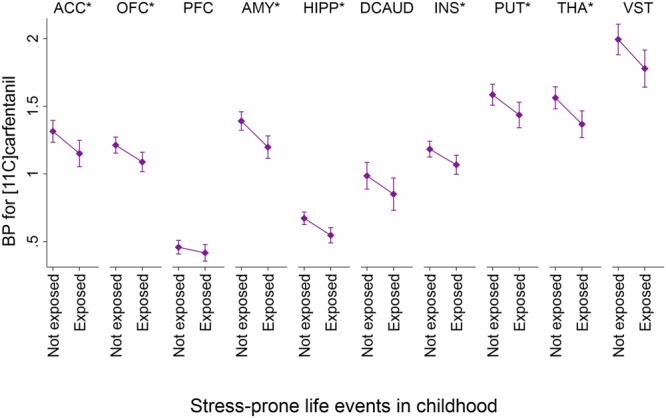
Plots of distributions of regional binding potentials for [^11^C]carfentanil in adulthood for participants exposed *vs*. not exposed to stress-prone life events in childhood. Adjusted for age and sex. * Statistically significant after FDR correction. ACC Anterior cingulate cortex, AMY Amygdala, DCAUD Dorsal caudate, HIPP Hippocampus, INS Insula, MCC Medial cingulate cortex, OFC Orbitofrontal cortex, PUT Putamen, THA Thalamus, VST Ventral striatum.

Next, we investigated whether stress-prone childhood events, disadvantageous emotional family atmosphere, and adverse socioeconomic environment predict μ–opioid receptor availability in the ROIs. The results are shown in Table 2 and illustrated in Fig. 1. When adjusted for age and sex, participants with stress-prone life events in childhood had lower binding potential for [^11^C]carfentanil in the orbitofrontal cortex (B = −0.19, uncorrected *p* = 0.0005), hippocampus (B = −0.12, uncorrected *p* = 0.0046), putamen (B = −0.21, uncorrected *p* = 0.0031*)*, amygdala (B = −0.18, uncorrected *p* = 0.0039*)*, insula (B = −0.16, uncorrected *p* = 0.0025), thalamus (B = −0.20, uncorrected *p* = 0.00762, and anterior cingulate cortex (B = −0.23, uncorrected *p* = 0.0022), dorsal caudate (B = −0.19, uncorrected *p* = 0.0326), and ventral striatum (B = −0.25, uncorrected *p* = 0.0153) in adulthood, when compared to participants without stress-prone life events. After FDR correction, all these associations survived except for the dorsal caudate and ventral striatum. Disadvantageous emotional family environment or adverse socioeconomic environment in childhood were not related to μ–opioid receptor availability in adulthood.

**Table 2:** The results of age- and sex-adjusted analyses, when investigating the differences in binding potential for [^11^C]carfentanil in the ROIs between participants with *vs*. without (a) stress-prone life events, (b) disadvantageous emotional family atmosphere, and (c) adverse socioeconomic environment in childhood.

	(a) Stress-prone life events (*n* = 37)			(b) Disadvantageous emotional family atmosphere (*n* = 38)			(c) Adverse socioeconomic environment (*n* = 39)		
	B	95% CI	Uncorrected *p* (FDR-corrected *p*)	B	95% CI	Uncorrected *p* (FDR-corrected *p*)	*B*	95% CI	Uncorrected *p* (FDR-corrected *p*)
ACC	−0.23	−0.36; −0.09	**0.0022 (0.0330)**^a^	0.04	−0.12; 0.19	0.6365 (0.8680)	0.00	−0.14; 0.15	0.9587 (0.9918)
OFC	−0.19	−0.29; −0.09	**0.0005 (0.0150)**^a^	0.05	−0.07; 0.16	0.4398 (0.8246)	0.00	−0.11; 0.12	0.9354 (~ 1.00)
PFC	−0.07	−0.16; 0.01	0.0987 (0.2961)	0.02	−0.07; 0.11	0.6381 (0.8323)	0.00	−0.08; 0.08	0.9772 (0.9772)
AMY	−0.18	−0.30; −0.06	**0.0039 (0.0234)**^a^	−0.05	−0.08; 0.18	0.4242 (0.8484)	−0.04	−0.16; 0.08	0.4949 (0.7814)
INS	−0.16	−0.26; −0.06	**0.0025 (0.0250)**^a^	0.08	−0.03; 0.19	0.1273 (0.3472)	−0.01	−0.11; 0.09	0.8374 (0.9662)
HIPP	−0.12	−0.20; −0.04	**0.0046 (0.0230)**^a^	−0.01	−0.10; 0.08	0.7494 (0.8993)	0.03	−0.06; 0.11	0.5060 (0.7590)
PUT	−0.21	−0.34; −0.08	**0.0031 (0.0233)**^a^	0.09	−0.06; 0.24	0.2204 (0.4723)	−0.02	−0.16; 0.11	0.7252 (0.9065)
THA	−0.20	−0.34; −0.06	**0.0072 (0.0309)**^a^	0.06	−0.09; 0.21	0.4426 (0.7811)	−0.01	−0.15; 0.13	0.8848 (0.9831)
DCAUD	−0.19	−0.36; −0.02	**0.0326 (0.1087)**	0.04	−0.13; 0.22	0.6172 (0.8817)	−0.06	−0.22; 0.10	0.4533 (0.7555)
VST	−0.25	−0.44; −0.05	**0.0153 (0.0574)**	0.14	−0.06; 0.34	0.1573 (0.3933)	−0.11	−0.30; 0.07	0.2190 (0.5054)

Bold *p* values were statistically significant at *p* < 0.05 before FDR correction.

*ACC* anterior cingulate cortex, *AMY* amygdala, *DCAUD* dorsal caudate, *HIPP* hippocampus, *INS* insula, *MCC* medial cingulate cortex, *OFC* orbitofrontal cortex, *PUT* putamen, *THA* thalamus, *VST* ventral striatum.

^a^Statistically significant at *p* < 0.05 after FDR correction.

Sensitivity analyses including age, sex, Harm Avoidance, and attachment style as covariates (*n* = 33–35) are reported in Supplementary Table 1. Before applying FDR correction, the effect of stress-prone life events on binding potential for [^11^C]carfentanil was significant in the anterior cingulate cortex (uncorrected *p* = 0.0106), orbitofrontal cortex (uncorrected *p* = 0.0041), amygdala (uncorrected *p* = 0.0280), insula (uncorrected *p* = 0.0207), putamen (uncorrected *p* = 0.0207), thalamus (uncorrected *p* = 0.0322), and ventral striatum (*p* = 0.0489). None of the associations, however, survived after FDR correction.

## Discussion

While animal studies have reported an association between environmental enrichment and enhancement of neurotransmitters such as noradrenaline and dopamine in mice and rats [46], this is the first prospective study to show an association with childhood family environment and regulation of the opioid system in humans. When compared to individuals not exposed to stress-prone life events, individuals who had exposed to **s**tress-prone life events in childhood had lower μ-opioid receptor availability in the orbitofrontal cortex, hippocampus, putamen, amygdala, insula, thalamus, anterior cingulate cortex, and dorsal caudate in adulthood. Thus, exposure to environmental instability such as normal-life stress-prone life events may have long-term influences on the opioid system, lasting into adulthood. Instead, adverse socioeconomic environment and disadvantageous emotional family environment (in terms of e.g. child-rearing attitudes or parental life satisfaction) were not longitudinally associated with μ-opioid receptor availability in adulthood.

Stress-prone life events were associated with a dysregulated mu opioid receptor binding. Previously, lower binding with the radiotracer [^11^C]carfentanil has been observed during a sustained painful state [47], during pain-induced affective responses [11], and in participants with subclinical anxiety [48]. There is evidence (mostly from animal studies) that the endogenous opioid system may modulate the effects of potentially distressing experiences: whether they lead to elevated neuroendocrine or autonomic stress responses in the long run, or not [47, 49]. In this way, the opioid system may help alleviating most painful states during exposure to stressors. Chronic stressors, however, may change the regulation of the opioid system under stress (including μ-opioid receptors) [49] that, in turn, may result in stress-related disorders [49]. Relatedly, the effect of stress-prone life events to became non-significant after controlling for Harm Avoidance, implying a potential mediating role of temperament-based susceptibility to stress. To sum up, our findings propose a tentative question whether exposure to stress-prone life events in childhood may result in long-term changes in the regulation of the opioid system.

A crucial region where stress-prone life events were linked to μ-opioid receptor availability was the orbitofrontal cortex. This may indicate altered processing of social interaction since social laughing and being touched by one’s partner relate to endogenous opioid release in the orbitofrontal cortex [9, 10]. Also, the orbitofrontal cortex is involved in expressing maternal love and vigilant protectiveness at times of infant’s distress [50]. Additionally, the orbitofrontal cortex is activated during value-based decision-making, i.e., making decisions on the basis of subjectively perceived reward or pleasantness of possible outcomes [51]. Thus, childhood stress-induced dysregulation of the μ-opioid transmission in the orbitofrontal cortex may imply altered perceptions of reward and pleasantness of social interaction with close others. Consistent with this, our additional analysis showed that the association of stress-prone life events with mu opioid receptor availability turned to be non-significant after taking into account attachment style. This suggests that attachment-related neurobiological processes may serve as an intermediating factor.

Importantly, the present study does not allow for making causal conclusions about the association between childhood family environment and the opioid transmission. It is known that a μ-opioid receptor gene (OPRM1) interacts with parenting practices when predicting later psychosocial outcomes [18–20]. Thus, children with certain genetic susceptibilities in mu opioid receptor binding may be more sensitive to childhood adversities. Also, it is also possible that children with innately low opioid transmission may have more likely a “difficult temperament” that, in turn, may make them more susceptible to receive maltreatment from their parents.

Interestingly, normal-life differences in emotional atmosphere (including, e.g., child-rearing attitudes and parental life satisfaction) or socioeconomic adversities were not related to μ-opioid receptor availability. Our results align with a previous study that also found no association between retrospectively assessed childhood traumatic experiences (including emotional, physical, and sexual abuse, as well as emotional and physical neglect) and μ-opioid receptor availability [21]. Both samples, however, included participants with relatively low levels of stress-prone events or traumatic experiences. Thus, it remains to be investigated whether more severe emotional or socioeconomic adversities could account for differences in μ-opioid receptor availability.

Regarding limitations, as our sample was comparatively small (*n* = 37‒39 in the analyses), our dataset may not likely have had sufficient statistical power. Thus, the results must be treated as preliminary and require replication in larger datasets. A larger sample size could provide possibilities to investigate more sophisticated aspects of the associations; for example, whether the associations between a number of childhood risk factors and opioid transmission might be curvilinear, or whether some childhood stressors are more crucial than others. Second, our small dataset did not allow us to investigate the potential moderating roles of adulthood protective factors. Therefore, more research is needed on possible other individual factors (e.g., social support) that might buffer against alterations in the opioid transmission if having been exposed to stressful events. Third, the dataset was originally collected to examine the associations between brain opioid system and the temperament trait of Harm Avoidance (Tuominen et al. [22]). Thus, we invited all the participants with low/high Harm Avoidance who could be matched with each other with regard to age, sex, and educational level. Although we controlled for Harm Avoidance in the additional analyses, this sampling procedure may have caused some sort of bias to the results.

In conclusion, the quality of childhood family environment may have long-term influences on the opioid system that is known to play a crucial role in experiencing social reward during social interaction and affective pain after social exclusion. Exposure to normal-life stress-prone events (such as change of school) was found to predict lower μ-opioid receptor availability in vivo in the orbitofrontal cortex and other brain regions crucial for socioemotional processing. Instead, normal-life differences in emotional family atmosphere (e.g., child-rearing attitudes) or socioeconomic family environment were not related to the opioid system in vivo.

## Supplementary information

Supplementary Table 1

## Data Availability

The dataset comprises health-related participant data, and their use is therefore restricted under the regulations on professional secrecy (Act on the Openness of Government Activities, 612/1999) and on sensitive personal data (Personal Data Act, 523/1999, implementing the EU data protection directive 95/46/EC). Due to these legal restrictions, the data from this study cannot be stored in public repositories or otherwise made publicly available. However, data access may be permitted on a case by case basis upon request. Data sharing outside the group requires a data-sharing agreement. Investigators can submit an expression of interest to Prof. Jarmo Hietala, University of Turku, Finland, jahi@utu.fi.
