# Supplementary material for: Childhood family environment and μ-opioid receptor availability in vivo in adulthood
Source: Neuropsychopharmacology. 2025 Jan 31;50(7):1130–5. doi: 10.1038/s41386-025-02059-6 (PMC12089380; doi:10.1038/s41386-025-02059-6)
Supplement: Supplementary file 1 — Supplementary Table 1 [file 41386_2025_2059_MOESM1_ESM.docx]

**Supplementary Table 1.** The results of analyses, when investigating the differences in binding potential for [^11^C]carfentanil in the ROIs between participants with stress-prone life events, disadvantageous emotional family atmosphere, and adverse socioeconomic environment (vs. participants without these unfavorable environmental factors in childhood). Adjusted for age, sex, Harm Avoidance, and attachment style.

|  | (a) Stress-prone life events  (*n* = 33) | | |  | (b) Disadvantageous emotional family atmosphere (*n* = 34) | | |  | (c) Adverse socioeconomic environment  (*n* = 35) | | |
| --- | --- | --- | --- | --- | --- | --- | --- | --- | --- | --- | --- |
|  | B | 95 % CI | Uncorrected *p* value  (FDR-corrected *p* value) |  | B | 95 % CI | Uncorrected *p* value  (FDR-corrected *p* value) |  | B | 95 % CI | Uncorrected *p* value  (FDR-corrected *p* value) |
| ACC | -0.22 | -0.38; -0.06 | 0.0106 (0.1590) |  | 0.06 | -0.12; 0.23 | 0.5005 (9.8342) |  | -0.01 | -0.17; 0.15 | 0.9329 (0.9651) |
| OFC | -0.19 | -0.32; -0.07 | 0.0041 (0.1230) |  | 0.07 | -0.07; 0.21 | 0.2858 (0.5044) |  | 0.00 | -0.13; 0.13 | 0.9846 (0.9846) |
| PFC | -0.06 | -0.16; 0.03 | 0.1773 (0.4092) |  | 0.01 | -0.08; 0.11 | 0.7803 (0.8670) |  | 0.02 | -0.07; 0.10 | 0.7134 (0.8561) |
| AMY | -0.16 | -0.30; -0.02 | 0.0280 (0.1680) |  | 0.08 | -0.07; 0.22 | 0.2833 (0.5666) |  | -0.04 | -0.17; 0.09 | 0.5151 (0.8133) |
| INS | -0.14 | -0.26; -0.02 | 0.0207 (0.2070) |  | 0.11 | -0.01; 0.23 | 0.0678 (0.2543) |  | -0.01 | -0.13; 0.10 | 0.8238 (0.8826) |
| HIPP | -0.08 | -0.17; 0.01 | 0.0827 (0.2481) |  | 0.01 | -0.08; 0.11 | 0.7537 (0.8697) |  | 0.02 | -0.07; 0.10 | 0.6585 (0.8980) |
| PUT | -0.20 | -0.36; -0.03 | 0.0207 (0.1553) |  | 0.13 | -0.04; 0.30 | 0.1291 (0.3521) |  | -0.03 | -0.19; 0.13 | 0.7042 (0.8803) |
| THA | -0.18 | -0.34; -0.02 | 0.0322 (0.1610) |  | 0.09 | -0.08; 0.26 | 0.2838 (0.5321) |  | -0.03 | -0.19; 0.13 | 0.6914 (0.9018) |
| DCAUD | -0.18 | -0.38; 0.02 | 0.0814 (0.2713) |  | 0.05 | -0.16; 0.26 | 0.6252 (0.8931) |  | -0.05 | -0.24; 0.14 | 0.5664 (0.8496) |
| VST | -0.24 | -0.48; -0.00 | 0.0489 (0.2096) |  | 0.17 | -0.07; 0.41 | 0.1558 (0.3895) |  | -0.13 | -0.35; 0.09 | 0.2468 (0.5289) |
| Note: ACC = anterior cingulate cortex; AMY = amygdala; DCAUD =dorsal caudate; HIPP = hippocampus; INS = insula; MCC = medial cingulate cortex; OFC = orbitofrontal cortex; PUT = putamen; THA = thalamus; VST = ventral striatum. | | | | | | | | | | | |
